# Efficacy of Electrocuting Devices to Catch Tsetse Flies (Glossinidae) and Other Diptera

**DOI:** 10.1371/journal.pntd.0004169

**Published:** 2015-10-27

**Authors:** Glyn A. Vale, John W. Hargrove, N. Alan Cullis, Andrew Chamisa, Stephen J. Torr

**Affiliations:** 1 South African Centre for Epidemiological Modelling and Analysis, University of Stellenbosch, Stellenbosch, South Africa; 2 Natural Resources Institute, University of Greenwich, Chatham, United Kingdom; 3 Early Warning Systems, Wembley, Pietermaritzburg, Kwazulu-Natal, South Africa; 4 Division of Tsetse Control, Harare, Zimbabwe; 5 Liverpool School of Tropical Medicine, Liverpool, United Kingdom; 6 Warwick Medical School, University of Warwick, Coventry, United Kingdom; Universidad de Buenos Aires, ARGENTINA

## Abstract

**Background:**

The behaviour of insect vectors has an important bearing on the epidemiology of the diseases they transmit, and on the opportunities for vector control. Two sorts of electrocuting device have been particularly useful for studying the behaviour of tsetse flies (*Glossina* spp), the vectors of the trypanosomes that cause sleeping sickness in humans and nagana in livestock. Such devices consist of grids on netting (E-net) to catch tsetse in flight, or on cloth (E-cloth) to catch alighting flies. Catches are most meaningful when the devices catch as many as possible of the flies potentially available to them, and when the proportion caught is known. There have been conflicting indications for the catching efficiency, depending on whether the assessments were made by the naked eye or assisted by video recordings.

**Methodology/Principal Findings:**

Using grids of 0.5m^2^ in Zimbabwe, we developed catch methods of studying the efficiency of E-nets and E-cloth for tsetse, using improved transformers to supply the grids with electrical pulses of ~40kV. At energies per pulse of 35–215mJ, the efficiency was enhanced by reducing the pulse interval from 3200 to 1ms. Efficiency was low at 35mJ per pulse, but there seemed no benefit of increasing the energy beyond 70mJ. Catches at E-nets declined when the fine netting normally used became either coarser or much finer, and increased when the grid frame was moved from 2.5cm to 27.5cm from the grid. Data for muscoids and tabanids were roughly comparable to those for tsetse.

**Conclusion/Significance:**

The catch method of studying efficiency is useful for supplementing and extending video methods. Specifications are suggested for E-nets and E-cloth that are ~95% efficient and suitable for estimating the absolute numbers of available flies. Grids that are less efficient, but more economical, are recommended for studies of relative numbers available to various baits.

## Author Summary

With a view to refining studies of tsetse fly behaviour, we used a catch method, in the field in Zimbabwe, to assess and improve the efficiency of 0.5m^2^ electrocuting grids commonly used to sample tsetse that collide with netting while in flight or which alight on cloth. We found that the efficiency of both sorts of grid was improved if the ~40kV electricity supply pulsed at 1ms intervals, as opposed to the 5–15ms commonly used in the past. Efficiency was also improved if the energy per pulse was at least 70mJ. For the netting grid, which is ideally required to be invisible to the flies, the visibility seemed reduced if the netting were much finer than that used previously, and if the distance between the grid and its supporting frame were increased from 2.5cm to 27.5cm. The indications for muscoids and tabanids were much the same as for tsetse. Using these field results and a simple model of grid performance, we suggest the specification of grids suitable for various sampling purposes.

## Introduction

Since the early 1970s, electrocuting grids have been crucial in clarifying the behaviour of tsetse flies (Diptera: Glossinidae) and in informing the development of bait methods of sampling and controlling these insects [1,2]. Two main types of device have been used: (i) the electric net (E-net), consisting of a grid of fine wires over a sheet of fine black netting to intercept tsetse in flight, and (ii) the electric cloth (E-cloth) involving a grid over material on which tsetse alight. The devices have also proved useful against other Diptera, such as tabanids [3,4], muscoids [5], mosquitoes [6,7] and sandflies [8]. The devices are appealing because they can be more efficient than hand-net catching and can be operated in the absence of humans, whose presence confuses the interpretation of catch size and composition [9]. Moreover, the devices can be arranged in various ways to sample a wide range of behaviour, including attraction to baits from a distance, flying in various positions near them, alighting, feeding on hosts and entering traps [7,9]. However, it is important to maximize and measure the efficiency of the devices if their catches are to be used confidently to interpret the fine detail of insect behaviour and to predict the performance of baits employed for surveys and control [10].

The initial estimates of the efficiency were made with grids powered by transformers that took a DC current of 0.6A at 12V from a lead/acid battery and converted it to pulses of ~40kV that lasted for 0.18ms at intervals of 8.5ms, with 61mJ of energy per pulse [1]. From the number of tsetse caught by a grid 1m^2^, and the number seen flying away seemingly unharmed after touching it, it was estimated that the E-net and E-cloths caught 92% and 97%, respectively, of the tsetse making contact [1]. Unfortunately, the car radio vibrators used in the above transformers were not designed for the load they took in the circuitry, so they tended to burn out after a few hundred hours of use. This prompted the development of more durable, solid state transformers that could provide up to 210mJ per pulse, which was sufficient to give a vigorous spark in grids of up to 5m^2^. During the next two decades the pulse interval was commonly increased to 15–30ms, to prolong battery life, with no obvious loss in grid performance as judged by naked eye observations. However, the development of high speed video techniques in the late 1980s provided information more objective than that obtained with the naked eye. It emerged that the efficiency of E-nets at the longer pulse intervals could be as low as 40% if allowance were made not only for the failure of flies to be electrocuted on contact, but also for electrocuted flies not falling to the trays intended to collect them, and for tsetse seeing and avoiding the grids due to perception of the wires, netting or frames of the grids [11,12]. The main suggestions arising were that in order to maximize efficiency the visibility of the E-nets should be reduced and the pulse interval should be shortened to less than the 5ms that was as low as could be achieved reliably with the transformers then available. These suggestions could not be followed up at the time, since it was only in 2012 that it was possible to obtain large sheets of finer netting and transformers able to produce reliably the very short pulse intervals.

In considering ways of testing the finer netting and new transformers, it was recognized that while the video technique offers some important benefits, it is also subject to certain problems. For example, although it is believed possible to identify tsetse among the many sorts of insect videoed near baits in the field, it is impossible to identify the sex and species of tsetse and to classify all other insects. Methods based on catch comparisons would be relatively quick and cheap, and could allow production of data for a wide range of identified insects. Hence, present work developed catch methods of studying the efficacy of electrocuting grids for tsetse, muscoids and tabanids, and assessed the extent to which the results accorded with the indications of the earlier video work. The catch methods, in combination with a theoretical model of grid performance, were then used to gauge the effectiveness of the new transformers and finer netting.

## Methods

### Ethics

There were no ethical issues since all experimental objects were mechanical devices.

### Study area and test insects

All work was performed in the Mana Pools National Park of the Zambezi Valley of Zimbabwe, at Rekomitjie Research Station (16° 10' S, 29° 25' E, altitude 503m) where *Glossina morsitans morsitans* Westwood and *G*. *pallidipes* Austen occur. Study sites were in open woodland that provided dappled shade. Any tsetse, muscoid or tabanid caught in each experiment was recorded, but only tsetse were consistent components of the catches. The muscoid and tabanid groups were composed of many species, showing the diversity detailed by [3] and [5], respectively. The muscoid catches usually contained biting (Stomoxyine) and non-biting individuals (S1 Table, S2 Table). Where muscoids and tabanids are not discussed, their catches were too few to give reliable indications of treatment effects.

### Transformers and batteries

The transformers consisted of the latest type, and also the variety of old transformers used during the video work [11,12]. All were sold state and operated by initially transforming the 12V input to several hundred volts. That current was stored in a capacitor and then discharged into a car ignition coil to give the final output of ~40kV. The prime difference between the various circuits was the device used to produce the initial transformation. In the oldest circuits, detailed in [13], the device was a self-oscillating inverter running at ~50 Hz with a standard 12-0-12V to 220V mains (iron core) transformer. The energy and voltage of the output pulse varied according to component values and battery voltage. Energy per pulse ranged from 70–250mJ; pulse interval could not be less than 20ms (50Hz). The new circuit (S1 Fig) used a ferrite core transformer for the inverter. This oscillated at 25–30 kHz to charge a bank of capacitors very quickly, enabling the high voltage output to pulse at intervals as low as 1ms (1kHz). As the efficiency of this inverter was higher, it reduced the drain on the battery. Various capacitor values were used to regulate the energy per pulse, but to prevent circuit overload the maximum energy per pulse at pulse intervals of 1ms, 2ms and 5ms was 35mJ, 70mJ and 105mJ, respectively. The car coil was upgraded to a high quality sports type, so minimising the risk of breakdown. A fan was incorporated to cool all components and ensure maximum reliability. Batteries for all transformers were of the lead/acid type, of 50–100A/h capacity, and were fully charged at the start of sampling sessions.

The pulse interval at various settings of the transformers was initially measured by an oscilloscope in the laboratory. The correctness of the settings was confirmed in the field with the aid of a clock-work gramophone. A sheet of carbon paper was placed over the earthed metal turntable and a steel needle was fixed 5mm above the paper, so that the turntable and paper spun under it. The needle was connected to the transformer output; sparks passed through the paper to the turntable, punching small holes that were clearly visible when the paper was held to light. Pulse interval was calculated from the rotation speed of the turntable and the angular separation between holes.

Unless stated otherwise, transformers were set to pulse at intervals of 15ms, at an energy of 175mJ per pulse. This was called the standard setting.

### Grids and baits

**Fig 1:**
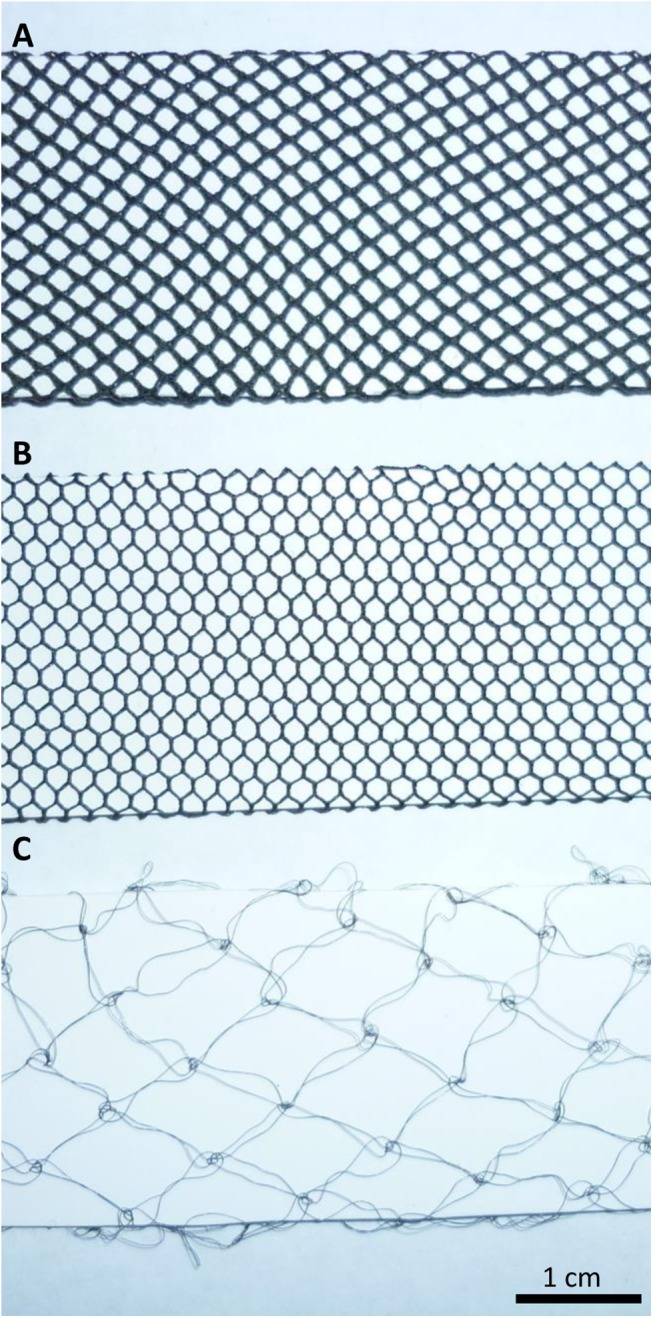
Types of netting incorporated into E-nets. A: coarse netting normally used for insecticide-treated targets. B: fine netting usually employed in E-nets for behavioural studies. C: ultra-fine netting used in some of the present work.

**Fig 2:**
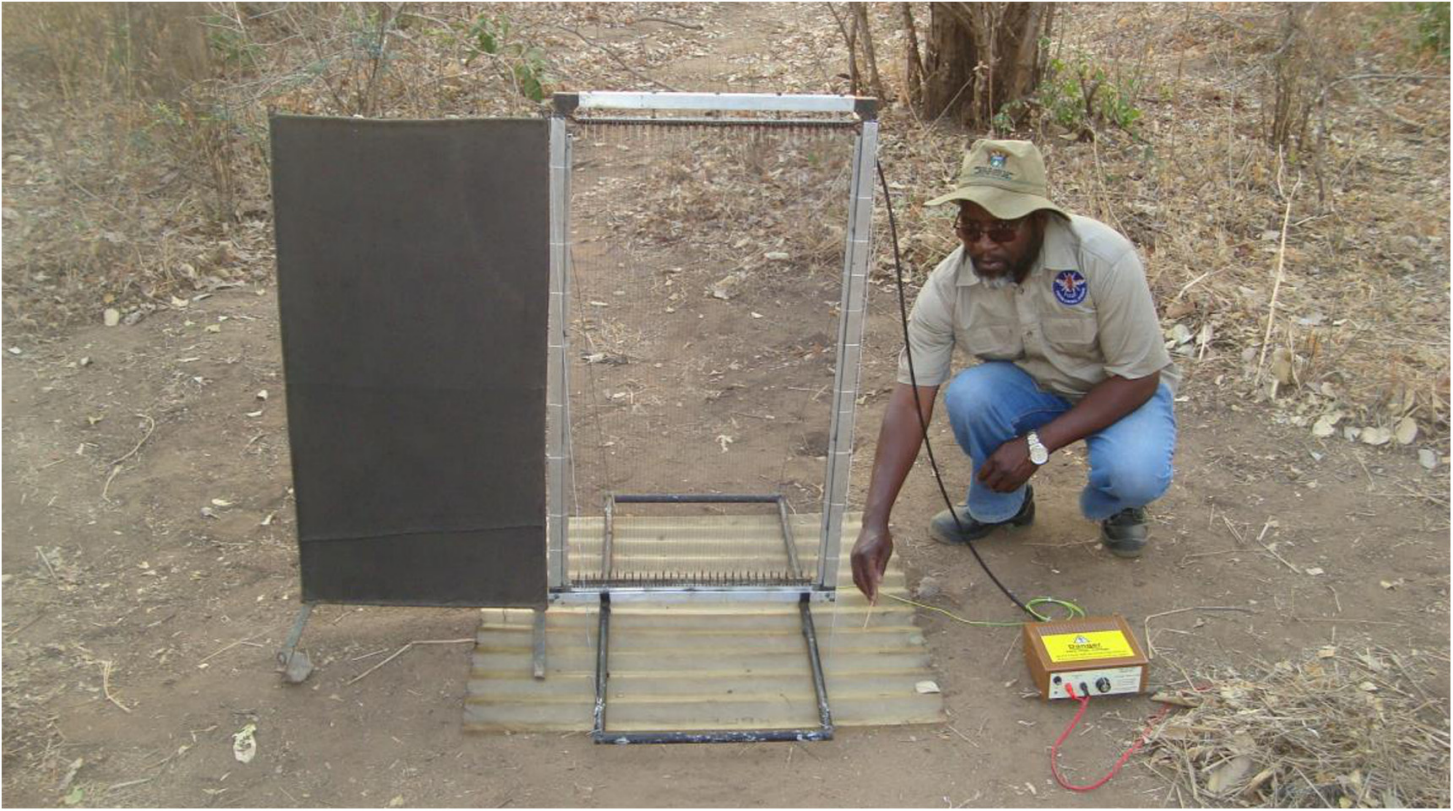
E-net by cloth. The yellow labelled box is the transformer, which was powered via the red leads from a battery hidden at the bottom right in a small pit covered by leaf litter. The black lead to the top of the E-net carried the high voltage output, and the yellow/green lead is the earthed return. A mirror image of this arrangement occurred 1.5m or 200m to the left, for the one-site or two-site methods, respectively.

Grids were suspended in frames made from aluminium square-tube, 2.5 x 2.5cm in cross-section. Unless stated otherwise the vertical and horizontal struts of the frame were 2.5cm from the nearest part of the grid and were unpainted. All grids consisted of copper wires that were 0.2mm in diameter, unvarnished, blackened by oxidation, and arranged vertically, with alternating charged and earth wires 8mm apart. The standard E-net comprised a double bank of wires, one on each side of a sheet of non-shiny, black polyester netting placed between them, and 6mm from each grid. Unless stated otherwise, the netting was of about 75% translucence (Fig 1B) (Raschel Warp Knit PD046/1060, Waverly Ltd, Zimbabwe), and was the same as that which has been used in most behavioural studies at Rekomitjie, including the video work [11,12]. However, since this fine netting is readily damaged and is a poor medium for insecticide deposition, the netting used to make targets routinely deployed in tsetse control operations is a little coarser (Fig 1A) (Fly Fence, Vestergaard SA, Switzerland). This coarser netting was sometimes used in present work. The very finest netting (Fig 1C) (Fynet 820, Lion Hair Care Ltd, UK) was the newly sourced material; it is normally used for cosmetic hair-nets. For E-cloth the netting was replaced by a sheet of non-shiny, black cotton cloth intended as an alighting stimulus. All grids were 100cm tall x 50cm wide. Flies were attracted to the close vicinity of the grids by odours consisting of acetone at 500mg/h, 1-octen-3-ol at 0.4mg/h, 4-methyl phenol at 0.8mg/h and 3-*n*-propyl phenol at 0.1mg/h, dispensed by the methods of [14]. Flies falling after electrocution became stuck in trays of corrugated fibreglass, extending 40cm from the base of the grid frame, with the polybutene sticky material being deposited only in the depressions (Fig 2). Such placement of the sticky deposit was to ensure that flies alighting on the tray, as against falling to it, had a reduced chance of becoming stuck, since alighting flies usually sat on the ridges.

### Experimental design

**Fig 3:**
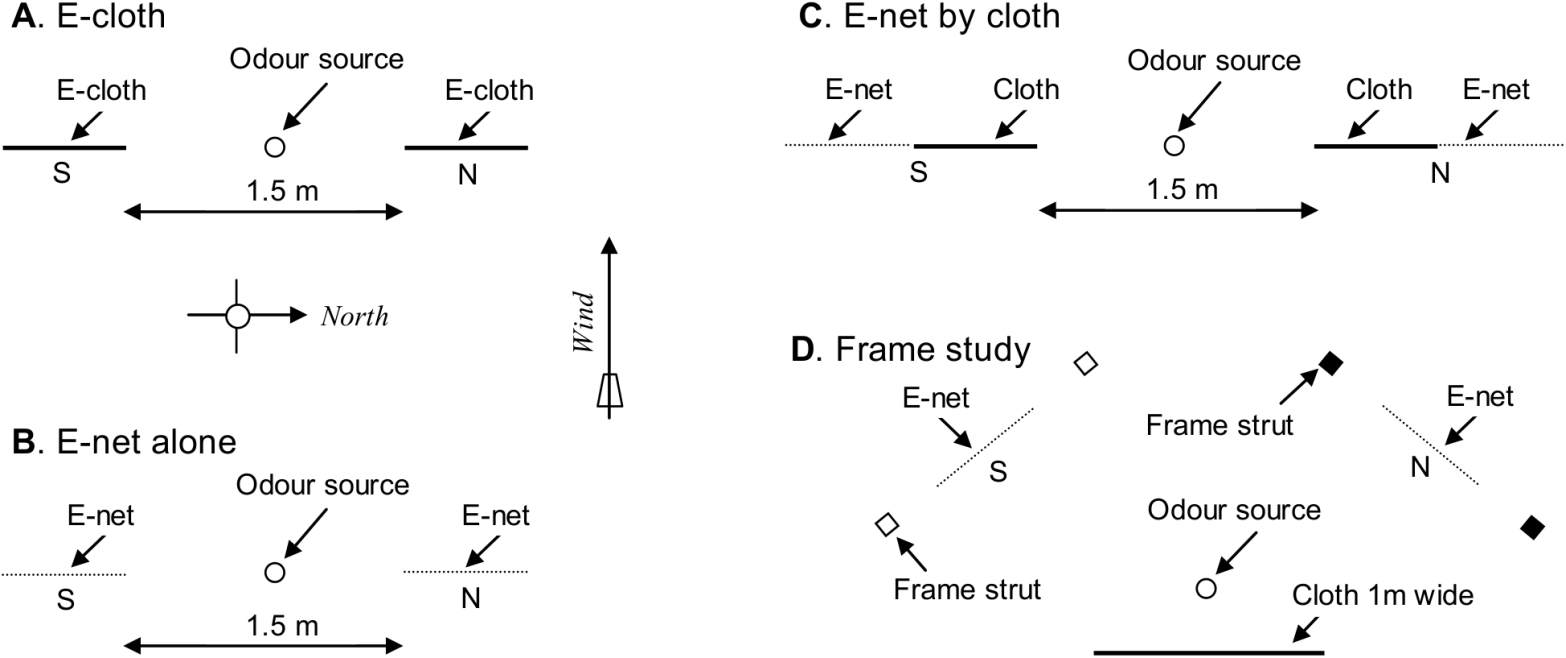
Plan views of the one-site arrangement of grids. A: E-cloth grids. B: E-net grids used without cloth nearby. C: E-net grids placed beside cloth. D: E-net grids, with various frames, placed across the approach routes to a cloth bait.

Some experiments used what was called the "one-site" method. For this, two separate treatments were employed, one in each of two nearby positions (N and S) 1.5m apart, although their precise arrangement varied between experiments (Fig 3). One treatment in each experiment was a control, consisting of a standard E-net or E-cloth operated at pulse intervals of 15ms, and the other was a test treatment involving an E-net or E-cloth that matched the control in all features except those under investigation. Catches with each treatment were made for 2–16 days in the last 3h before sunset, with the control and test treatments being alternated between N and S every 15min. On one day the test treatment started at the S position, and the next day at the N. Chi-squared tests assessed the probability for the hypothesis that the true distribution of the total catch was 50:50 between the control and test grid, implying that the test treatment was as effective as the control, *i*.*e*., that the test treatment gave a catch of 100% relative to the control. The term significant implies P<0.05. The 95% confidence limits (CL) of the test catch as a percent of the standard were calculated using the BinomHigh and BinomLow add-ins of Microsoft Excel.

One objection to the one-site method is that insects which fail to be captured on first contact with one treatment might then go to the other treatment to be caught, so accentuating the catch differences between contrasting treatments. This would tend to understate or overstate the electrocuting efficiency of treatments that are respectively less or more effective than the control. Such a potential problem has been recognized as applying also in the video work [11], but it was not possible to test its importance then. To assess its importance in present work, catches were made by the "two-site" method, in which the distance between the control and test treatments was increased from the 1.5m shown in Fig 3 to 200m, so that each treatment was effectively at a different site. Each of the two sites was provided with its own odour source, located just beyond the West edge of the sticky tray. A single treatment was operated at one of the sites for the whole 3h sampling period of each day, so that if a fly failed to be electrocuted on first contact with that treatment it was less likely to go to the other site to be caught. Experiments of this type reallocated the sites for each treatment from day to day, following a random cross-over design, with an analysis of variance performed on daily catches transformed to log(n+1). After detransforming, the mean catch with the test treatment was then expressed as a percent of the control mean.

## Results

### Comparison of one-site and two-site methods

The one-site and two-site methods were each employed to assess the performance of pulse intervals of 1ms relative to the 15ms control, in separate experiments that used either E-cloth alone, E-nets alone, or E-nets beside cloth (Fig 2). Each experiment comprised three to four sessions of four days each, distributed fairly evenly with the period September 2012 to March 2013. Since the data of each experiment gave no reason to suppose that the distribution of insects between the East and West sides of the devices was affected significantly by pulse interval (S1 Table), the data for the East and West sides were pooled for further analysis.

With the one-site method (Table 1), as with the two-site method (Table 2) the observed catches at 1ms pulse intervals were almost always greater than those at the control interval of 15ms. Moreover, the observed differences were roughly similar on average, with the catches at 1ms intervals being 141% of the control when the one-site method was used, as against 153% for the two-site method. These results offer *prima facie* evidence that the one-site method does not inflate materially the apparent performance of the more effective treatment. However, the confidence limits of the one-site method were relatively tight, so that ten of the results with this method showed catches significantly greater than the control, as against only three with the two-site method.

**Table 1:** Comparison of catches with pulse intervals of 1ms and 15ms, using the one-site method.

Experiment and test insect	Total catch		Percent catch	
	1ms	15ms	Mean	95% CL
**Expt. 1. Net alone, 16 replicates,**				
*G*. *m*. *morsitans* males	24	22	109	59–204
*G*. *m*. *morsitans* females	12	9	133	52–358
*G*. *pallidipes* males	27	18	150	80–289
*G*. *pallidipes* females	71	48	148*	101–218
Total tsetse	134	97	138*	106–181
Muscoids	223	129	173*	139–216
Tabanids	6	6	100	27–374
**Expt. 2. Net by cloth, 12 replicates**				
*G*. *m*. *morsitans* males	45	27	167*	101–279
*G*. *m*. *morsitans* females	29	25	116	66–207
*G*. *pallidipes* males	32	23	139	79–249
*G*. *pallidipes* females	92	56	164*	117–233
Total tsetse	198	131	151*	121–190
Muscoids	864	427	202*	180–228
Tabanids	323	272	119*	101–140
**Expt. 3. Cloth alone, 16 replicates**				
*G*. *m*. *morsitans* males	95	90	106	78–142
*G*. *m*. *morsitans* females	34	30	113	67–192
*G*. *pallidipes* males	24	21	114	61–216
*G*. *pallidipes* females	42	19	221*	126–402
Total tsetse	195	160	122	98–151
Muscoids	255	148	172*	140–212
Tabanids	6	6	100	27–374

The mean percent catch is the total catch at pulse intervals of 1ms, as a percent of the total at 15ms. Energy per pulse was 35mJ at all intervals. An asterisk next to the mean percent catch indicates that the catch with 1ms intervals differed from that with 15ms intervals, at P<0.05.

**Table 2:** Comparison of catches with pulse intervals of 1ms and 15ms, using the two-site method.

Experiment and test insect	Mean daily catch		Percent catch	
	1ms	15ms	Mean	95% CL
**Expt. 1. Net alone, 16 replicates**				
*G*. *m*. *morsitans* males	1.12	0.74	152	32–360
*G*. *m*. *morsitans* females	0.51	0.34	150	7–360
*G*. *pallidipes* males	1.40	0.58	241	83–498
*G*. *pallidipes* females	1.67	0.90	186	64–394
Total tsetse	5.22	2.85	183	89–347
Muscoids	9.32	4.94	189*	131–269
Tabanids	0.29	0.17	174	51–319
**Expt. 2. Net by cloth, 12 replicates**				
*G*. *m*. *morsitans* males	4.31	3.10	139	84–221
*G*. *m*. *morsitans* females	4.29	2.88	149	77–268
*G*. *pallidipes* males	12.15	11.22	108	83–140
*G*. *pallidipes* females	30.45	20.62	148	96–225
Total tsetse	54.64	40.43	135*	106–171
Muscoids	68.43	40.13	171	97–298
Tabanids	11.60	5.78	201	85–449
**Expt. 3. Cloth alone, 16 replicates**				
*G*. *m*. *morsitans* males	4.70	4.24	111	64–182
*G*. *m*. *morsitans* females	1.52	2.04	75	31–141
*G*. *pallidipes* males	3.75	2.36	159	90–265
*G*. *pallidipes* females	5.07	4.87	104	54–187
Total tsetse	17.53	15.40	114	76–169
Muscoids	10.46	4.61	227*	155–327
Tabanids	0.80	0.80	100	57–153

The mean percent catch is the mean daily catch at pulse intervals of 1ms, as a percent of the mean daily catch at 15ms. Energy per pulse was 35mJ at all intervals. An asterisk next to the mean percent catch indicates that the catch with 1ms intervals differed from that with 15ms intervals, at P<0.05.

Hence it seemed that the one-site and two-site methods showed basically the same sort of thing, but that the one-site method was the more precise. Given that it is also easier to run one site as against two, all further work used the one-site method. Moreover, since the sex and species of tsetse showed no clear differences in their susceptibilities to different electrocuting systems, as illustrated in Tables 1 and 2, the data for each sex and species are pooled in further reporting.

### Electrical variations

#### Need for electricity

The interpretation of the catches of the electrocuting devices assumes that the flies stuck in the trays arrived there as a result of electrocution. However, it is commonly observed that some clumsily-flying insects, such as beetles, often fall to the trays after colliding with non-electrified grids, and various types of insect occasionally get stuck after alighting on the trays. To check the extent to which these matters apply to tsetse, catches from grids with standard electrification were compared with those from grids that were not electrified. Using the E-cloth, seven tsetse were caught without power, as against 310 with power. For the E-net the figures were nil and 215, respectively. This suggests that the vast majority of tsetse stuck below electrified grids were caught due to electrocution.

#### Pulse frequency and energy

**Fig 4:**
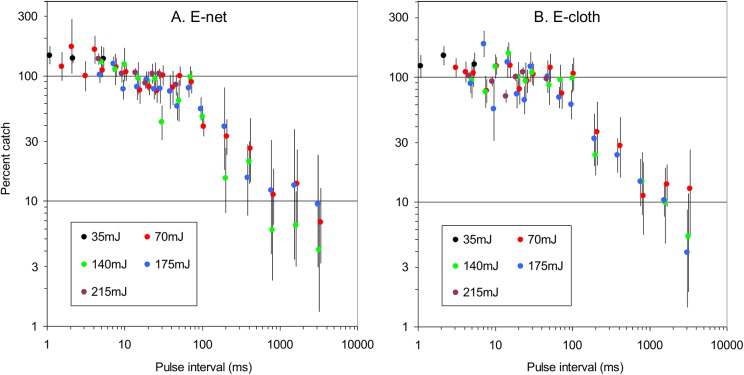
Effect of pulse interval on catches of tsetse from grids. A: E.net grid beside cloth. B: E-cloth. Catches at each interval tested are expressed as a percent of simultaneous catches from a control grid operating at a pulse interval of 15ms. Separate comparisons are made at various energy levels (mJ) per pulse, and the results are plotted separately. The test and the control treatments involved in any one plot had the same energy per pulse. Vertical bars through plots indicate the 95% confidence limits of the mean percent. Plots are often displaced a little horizontally to ensure that the confidence limits of the each plot can be distinguished.

In a series of experiments with E-net and E-cloth grids the catches at various pulse intervals were compared with those using the 15ms standard. The energy per pulse varied between experiments but within experiments it was always the same for the standard and test frequencies. The results (Fig 4, S2 Table) for each type of grid showed that pulse energy had no clear impact on the effects of pulse frequency. Given the confident limits of the plots, there is some latitude in fitting curves to the frequency data. However, it does seem that the most appropriate curves are complex. Thus, with the E-net (Fig 4A), the catches started to decline when pulse frequency increased above 1ms, but remained roughly level between 15ms and 70ms, before declining sharply to 3200ms. With the E-cloth (Fig 4B) the results were similar except that the catch at intervals of around 1ms seemed hardly greater than 100%, and the section of roughly level catches seemed more extensive, occurring between 5ms and 100ms.

Although the data for muscoids and tabanids were less complete (S2 Table), it was clear that pulse interval was important with these flies. This is illustrated by the pooled data for various groups of pulse intervals. Thus, at intervals of 1–4ms the percent catches with the E-net grid were high, at 160.2% (total control catch = 2555) for muscoids and 113.6% (508) for tabanids, as against percent catches of only 20.8% (2789) and 31.8% (115), respectively, at intervals of 200–3200ms. With the E-cloth the percent catches for tabanids and muscoids at 1–4ms intervals were 138.0% (1643) and 102.5% (159), respectively, compared to 14.8% (9367) and 31.8% (286), respectively, at 200–3200ms.

#### Further study of energy

Although the above work showed that there was no marked interaction between energy per pulse and pulse frequency, the work did not assess the effect of energy *per se*. That effect was investigated by comparing the efficacy of energies of 35–215mJ at pulse intervals of 15ms. The results (Table 3) show that the only significant effects were reductions in catches at the very lowest energy, *i*.*e*., 35mJ per pulse. Since that low energy was used primarily to ensure that pulse intervals as low as 1ms could be used without causing the transformers to malfunction, it was pertinent to assess whether it is better to use short pulse intervals and low energy per pulse, or longer intervals and higher energy. Such assessments (Table 4) indicated that catches of tsetse at the E-net were enhanced at the short pulse interval, despite the energy per pulse being low. For muscoids at the E-cloth the energy per pulse seemed more important than pulse frequency. In other cases catches were similar at all energy and frequency combinations.

**Table 3:** Test catches at various energies per pulse, compared to control catches at an energy of 175mJ.

Insect	Grid	Test energy	Total catch		Percent catch	
			Test	Control	Mean	95% CL
Tsetse	E-Net	35mJ	168	274	61*	50–75
		70mJ	164	164	100	80–125
		215mJ	417	467	89	78–102
	E-Cloth	35mJ	139	186	75*	60–94
Muscoids	E-Net	35mJ	184	479	38*	32–46
		70mJ	19	26	73	38–137
		215mJ	49	36	136	87–215
	E-Cloth	35mJ	88	223	39*	30–51
Tabanids	E-Net	35mJ	65	117	56*	40–76

The mean percent catch is the total catch at the test energy, as a percent of the total at the control. All pulses intervals were 15ms. An asterisk next to the mean percent catch indicates that the test and control catches differed at P<0.05.

**Table 4:** Test catches at short pulse intervals and low energy per pulse, compared to control catches at longer intervals and higher energy.

Grid	Insect	Total catch		Percent catch	
		Test	Control	Mean	95% CL
Tsetse	E-Net	1768	1451	122*	114–131
	E-Coth	281	251	112	94–133
Muscoids	E-Net	928	942	99	90–108
	E-Cloth	69	97	71*	51–98

The test conditions were pulse intervals of 1ms and energy of 35mJ per pulse, as against control conditions of 15ms and 175mJ. The mean percent catch is the total catch at the test power, as a percent of the total at the control. An asterisk next to the mean percent catch indicates that the test and control catches differed at P<0.05.

### Variations to grid structure

#### Reduced visibility of E-net grids

The use of a double bank of wires, with netting between, may seem unnecessarily complex for catching tsetse in flight. If the netting could be deleted to leave just a double bank of wires, or if only one bank of wires would suffice, the visibility of the device could be reduced. Tests with grids simplified in these ways, and used at pulse intervals of 5–100ms, showed that the performance was reduced by around a quarter to a half by having just the net removed, so leaving a double bank of wires, or reduced even more when only one bank of wires was left (Table 5). Relatively few muscoids were caught in these experiments but they appeared to show roughly the same pattern as for tsetse. Thus, the pooled data for muscoids at all pulse intervals showed a relative catch of 48% (control catch 80, CL = 31–71) with the double bank, and 37% (57, 21–62) with the single bank. Naked eye observations of tsetse striking the non-netted grids showed that many flies passed right through seemingly unharmed—something never seen when netting was present. Thus, while present simplifications to grid structure might reduce the visibility of E-nets, any advantage of this seems greatly outweighed by a reduced efficiency of electrocution.

**Table 5:** Test catches of E-net grids with the netting removed and the wires forming a double or single bank, compared to control catches with a standard E-net.

Insect	Test wires	Total catch		Percent catch	
		Test	Control	Mean	95% CL
Tsetse	2 banks	888	1263	70*	64–77
	1 bank	586	2162	27*	25–30
Muscoids	2 banks	38	80	48*	31–71
	1 bank	21	57	37*	21–62

The mean percent catch is the total catch with the test wires, as a percent of the total at the control. In each comparison the pulse interval was 15ms and the energy per pulse was 175mJ. An asterisk next to the mean percent catch indicates that the test and control catches differed at P<0.05.

#### Visibility of netting

Over the last 30 years, several tests have been made of the catches from standard E-nets provided with fine netting, compared to such devices with the various types of coarser netting preferred for incorporation into insecticide-treated targets. In accord with the video evidence that the visibility of netting can induce tsetse to avoid E-nets [11,12], the tests suggested that the catches are reduced by about a third with the coarser netting. For example with the coarse netting shown in Fig 1A, the catch as a percent of the control catch with the fine netting of Fig 1B was 60% (control catch 546, CL 52–69) for tsetse, 84% (150, 66–107) for muscoids and 63.2% (106, 46–87) for tabanids.

The implication of these results seemed to be, as envisaged during the video work [12], that if the netting were ultra-fine, such as the netting of Fig 1C, then the catch of the E-nets would increase. In fact, however, the catches with a single layer of the ultra-fine netting were reduced by about a fifth or a third relative to the control catch with the fine netting of Fig 1B, whether the pulse interval used for the control and test treatment was 1ms or 15ms (Table 6). This result could have been caused by tsetse travelling straight through the ultra-fine netting, since the mesh size approached the wing span of tsetse. However, when the grid with ultra-fine netting was watched in the field, and when 100 tsetse were released about a metre from it in the laboratory, none was seen to pass through. Nevertheless, to ensure that through passage was impossible, a second round of studies was performed with three layers of the ultra-fine netting (Fig 1C) put together, to reduce the effective size of the mesh, while ensuring that the visibility was still substantially less than the fine net control (Fig 1B). As expected, the performance of the treble layer of ultra-fine netting was about the same as that of the single layer, confirming that passage through a single layer was not important.

**Table 6:** Test catches with E-nets incorporating various layers of ultra-fine netting, compared to control catches with a standard E-net, each operated at various pulse intervals.

Insect	Pulse interval	Test layers	Total catch		Percent catch	
			Test	Control	Mean	95% CL
Tsetse	1ms	1 layer	598	693	86*	77–96
		3 layers	748	899	83*	75–92
	15ms	1 layer	222	268	83*	69–99
		3 layers	173	259	67*	55–81
Muscoids	1ms	1 layer	702	1045	67*	61–74
		3 layers	141	188	75*	60–94
	15ms	1 layer	271	470	58*	49–67
		3 layers	20	30	67	36–121

The mean percent catch is the total catch with the test layers, as a percent of the total at the control. In any one comparison, the test and control E-nets were operated at the same pulse intervals, and always at 35mJ per pulse. An asterisk next to the mean percent catch indicates that the test and control catches differed at P<0.05.

#### Frame colour and location

It is known that tsetse can avoid flying near wooden poles of about the same thickness as the aluminium frames of present grids [15], and that they can be repelled by shiny objects [1]. To elucidate how these matters might bear on the optimal design of frame, studies were made with grids in which the vertical supporting struts of the frame were moved from their normal position of 2.5cm from the edges of the grids, to occur 27.5cm away, and with the frames at either distance being left in their normal shiny condition or painted with black PVA or yellow zinc chromate undercoat. Each of these paints gave a matt finish. The results (Table 7) showed no consistent effect of frame colour. However, the observed catches of tsetse, muscoids and tabanids as a percent of the standard were almost always increased when the distance between the grid and vertical struts was extended to 27.5cm. For tsetse and muscoids this increase in catch averaged 20%. For tabanids it was always much greater, averaging 140%, albeit that the total catches of tabanids were generally low, so that the indications for these insects are of reduced reliability.

**Table 7:** Test catches at E-nets with frames of various colour and different distances from the grids, compared to control catches using E-nets with standard frames.

Insect	Test frame		Total catch		Percent catch	
	Colour	Distance	Test	Control	Mean	95% CL
Tsetse	Aluminium	27.5cm	382	248	154*	131–181
	Black	2.5cm	346	256	135*	115–159
		27.5	305	214	143*	119–171
	Yellow	2.5cm	318	325	98	84–115
		27.5	362	334	108	93–126
Muscoids	Aluminium	27.5cm	1131	819	138*	126–151
	Black	2.5cm	898	660	136*	123–151
		27.5cm	1162	780	149*	136–163
	Yellow	2.5cm	591	467	127*	112–143
		27.5cm	284	224	127*	106–152
Tabanids	Aluminium	27.5cm	189	64	295*	221–399
	Black	2.5cm	113	81	140*	104–188
		27.5cm	206	93	222*	173–286
	Yellow	2.5cm	21	29	72	39–131
		27.5cm	15	7	214	82–621

The mean percent catch is the total catch with the test frame, as a percent of the total at the control. The control frame was aluminium colour, at a distance of 2.5cm. In each comparison the pulse interval was 15ms and the energy per pulse was 175mJ. An asterisk next to the mean percent catch indicates that the catch with the test frame differed from that with the control frame, at P<0.05.

### Interpretation of catches

#### Absolute efficiency

The efficiency of electrocuting devices can be regarded as depending on many sorts of event [1,11,12]. First, there are events which affect the number of tsetse that contact the grid. These involve flies colliding with the grid in apparent ignorance of its presence, or being attracted to the grid, or avoiding the grid by flying round it, over it or by executing a U turn. Next, there are the two main events that occur when the flies touch the grid: either the flies are electrocuted, or they fly away seemingly unscathed. Finally, the flies that are electrocuted either fall to the trays to stay there or to struggle free after a while; or the flies fall clear of the tray and so avoid being recorded as part of the catch. These matters are complicated by the fact that different researchers focus on different groups of events and measure them in different ways, using different energies and frequencies of pulse. For example, the video work involved simulated trays that collected tsetse falling within 40cm of the grids [11,12], whereas the early work performed with the naked eye used grids mounted in the back of a Land-Rover pick-up, with flies being collected rapidly after falling within ~1m of the grids [1]. Moreover, both types of work involved subjectivity. Thus, with the video studies the experimenter had to decide whether a fly that did not fall into the tray was unaffected, or would have fallen outside of the video field. With the grid on the Land-Rover, the numbers of flies seen to touch the screen and fly away unharmed may well have underestimated the true numbers doing so.

Much of the debate about the proportions of electrocuted flies that fall to the tray and remain there is somewhat irrelevant since that proportion can be improved by simply making the tray wider and coating it with sticky deposit [1]. A more fundamental and intriguing matter is the efficiency which insects are killed or stunned after contact, so that they become available to fall to a fully effective tray. The fact that the 40cm-wide trays used in the present work were unlikely to have caught all of the falling insects need not be particularly serious. This is because the interpretation of the data does not rely directly on the absolute catch with each test treatment, but rather on the catch relative to the control catch. Hence, assuming that the performance of the trays was about the same with the test treatments as it was with the control that used standard grids and 15ms pulse intervals, the observed relationships between catches can be taken as indices of the relationships between the absolute efficacies of electrocution. These indices can then be converted to estimates of absolute efficiency by reference to the video work [12] which showed that the absolute efficiency at pulse intervals of 15ms was ~ 0.72 for standard E-nets if electrocuted flies of all fates were pooled. For standard E-cloth the absolute efficiency at 15ms intervals was less clear but it was implied [12] that the figure of ~ 0.72 was again applicable.

**Fig 5:**
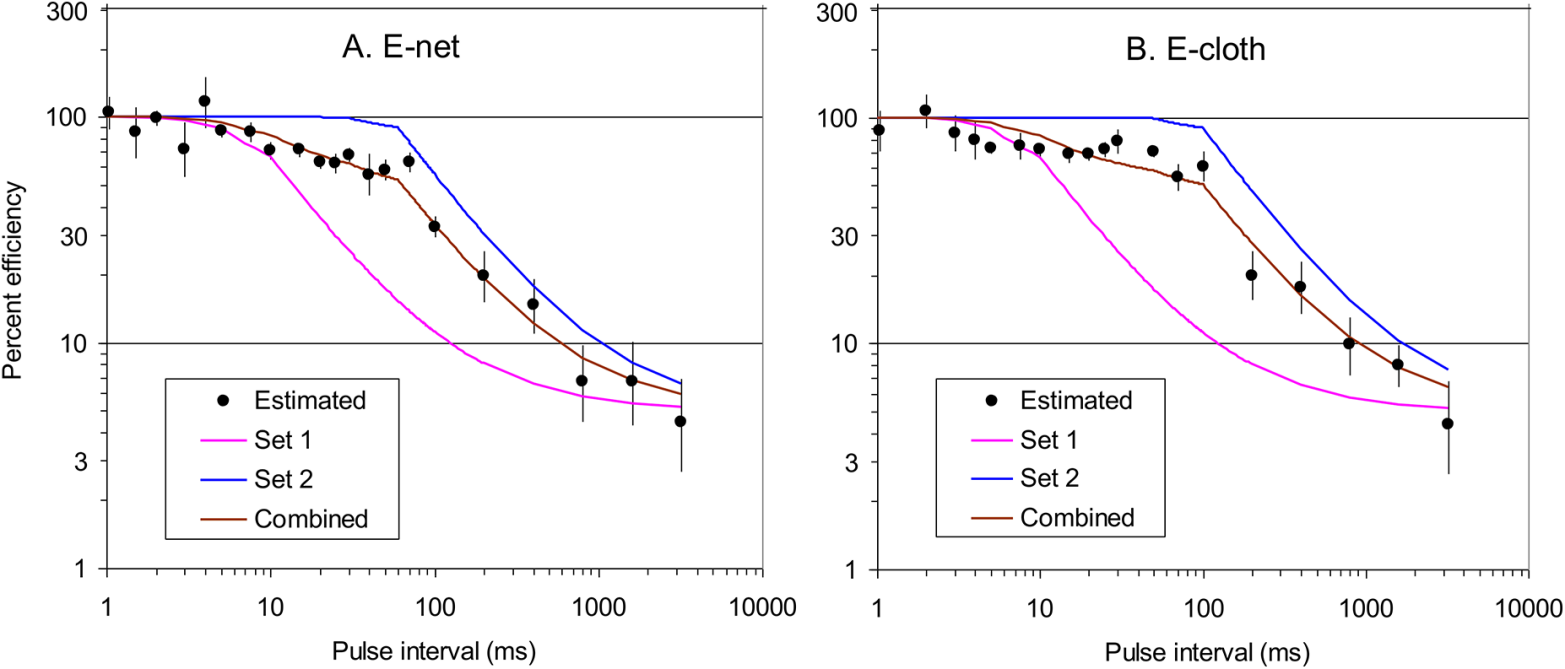
Fitting curves to the estimated absolute efficiency of electrocution of tsetse contacting grids operated at various pulse intervals. A: E.net grid beside cloth. B: E-cloth. The plots of percent absolute efficiency derive from pooling and adjusting the data for relative catches at various energies per pulse in Fig 4, as detailed in the text. Vertical bars through the plots indicate the 95% confidence limits. The curves labelled Set 1 and Set 2 refer to those modelled with the various sets of parameter values indicated in the text. The curve labelled Combined is the average of the Set 1 and Set 2 curves.

Based on the above principles of interpreting present catches, the estimates of absolute efficiency at various pulse intervals are as in Fig 5. To simplify that figure, all of the data for separate energies at each pulse interval have been pooled, consistent with the extensive overlaps evident in almost all of the confidence limits shown previously (Fig 4). The number of different lengths of pulse interval studied during the present work was 21, ranging from 1ms to 3200ms, as against only three different lengths in the video work, ranging from 5ms to 100ms [12]. Not surprisingly, therefore, present data expose greater detail for the relationship between catches and pulse interval. In trying to make sense of this relationship it is pertinent to consider a theoretical model, as below.

#### Model

It can be taken that about 5% of flies would have been caught even if the pulse interval were much greater than the maximum of 3200ms studied in present work. This is because a few flies are caught with non-electrified grids, and also because on a few occasions the flies become entangled in the grid and can stay there for many seconds—sometimes even resting on the net or cloth if the power is not turned on. For the remaining 95% of flies, suppose that the probability that a single pulse fails to kill or stun a fly is *q*, and assume that this value is independent of how many pulses the fly has experienced already. This means that if the fly experiences *k* pulses it survives with probability *q* ^*k*^ and, conversely, does not survive with probability 1—*q* ^*k*^. Now suppose that a fly spends a time *t* on the grid. The number of pulses that it experiences while there will be a function of *t* and of the pulse interval *s*. In general the expected number (*k*) of pulses that a fly experiences will be given by *k* = int[*t*/*s*], where the function "int" indicates that we are taking only the integer part of *t*/*s*. However, there will be a distribution between *k* and *k* + 1 pulses, where the proportion (*S*_k+1_) getting *k* + 1 pulses will be given by *S*_k+1_ = (*t*/*s*)—*k*, with *S*_*k*_ = 1- *S*_k+1_. So the probability (*Q*) that a fly survives is given by the probability that it receives *k* pulses, multiplied by the probability of surviving *k* pulses, plus the probability that it receives *k*+1 pulses, multiplied by the probability of surviving *k*+1 pulses. Hence:

$$Q=\;q^{k+1}\;S_{k+1}+q^{k}\;(1\;-\;S_{k+1})=q^{k}+S_{k+1}\;(q^{k+1}\;-\;q^{k})$$

The probability that the fly does not survive is 1 –*Q*.

In exploring various values for the parameters of the model it was impossible to find a single set of values that explained the data well, as exemplified by Sets 1 and 2 for the E-net in Fig 5A. For Set 1 it is taken that *q* = 0.33, and *t* = 10ms to correspond with the shortest contact time suggested by the video work [12]. The resulting curve fits well the data for pulse intervals of 1–10ms, but that curve fits poorly at around 15–400ms, mainly because it drops too rapidly in the 15–70ms range. For Set 2, in which *q* and *t* are changed to 0.1 and 60ms, respectively, a more level situation results in the 15–70ms range, but then the curve is too high at all pulse intervals above 5ms. The inability to explain the observed results by a single set of parameter values accords with the reasonable expectation and common observation that the contact which flies make with the grids is variable. For example, flies approach the grids at various angles [11] and can either strike a wire head on, or arrive between adjacent wires to make perhaps a longer and more effective contact between the charged and the earthed components. Presumably, the values of *t* and *q* for Set 1 of the E-net model might simulate a brief contact with a single wire, whereas the values of Set 2 simulate arrival between wires. In any event, if it is allowed that Set 2 contacts are as numerous as Set 1, the combined curve fits the E-net data tolerably well (Fig 5A). Likewise, the curve for E-cloth performance appears about right when the Set 1 and Set 2 values for this grid are the same as the those for the E-net, except that *t* is enhanced from 60ms to 100ms in Set 2 (Fig 5B).

## Discussion

To improve our knowledge of the efficacy of electrocuting methods of sampling tsetse, muscoids and tabanids we compared the catches from various devices electrified by pulses of high voltage at a range of frequencies and energies. We found that the relative catches from various electrocuting devices was not affected significantly by positioning the devices either 1.5m or 200m apart, but since the 1.5m separation was easier to manage and gave tighter confidence limits we adopted the 1.5m separation as standard for further work. Such work, combined with modelling the effect of pulse frequency, confirmed and extended what previous work with catch methods or video recording has indicated for the effects of pulse characteristics and grid visibility. Regarding pulse frequency, we concede freely that the inputs used in the modelling of E-net performance are somewhat arbitrary. The modelling is offered simply to show that the seemingly complex curve relating frequency and catches in present field work is credible.

Regarding visibility, the main concern is the extent to which flies are attracted to the grids or repelled by them. Since the E-cloth was designed specifically to attract flies to the cloth, it is not surprisingly that the video evidence is consistent with attraction and no repellence [12]. The situation is more complex with E-nets because video work shows flies avoiding the fine netting and wires of the grid, and also avoiding the frame [11,12]. Present catch methods confirm that frames can have an effect but were surprising in suggesting that the fine netting was attractive relative to the ultra-fine netting. Presumably this attractiveness is evident mostly for flies approaching from an acute angle, which makes the netting and wires more conspicuous. The fact that the attractiveness of the fine net seemed not to be noticed in the video work is perhaps explicable by the impossibility of distinguishing between a fly travelling straight to the netting because it has not seen it, or because it has. In any event, the present studies of the visibility of netting with savannah tsetse deserve extension to riverine and forest species of tsetse.

Four important points have emerged. First, present results and the video work concur in suggesting that the original naked-eye assessments of the efficiency of electrocution at 8.5ms, and the measured invisibility of E-nets, were flattering. In reality, the efficiency of electrocution at the pulse interval of 8.5ms seems around only 75%, not the 92–97% originally claimed [1]. Second present field work and the modelling supports the video evidence that a reduction in pulse interval can improve efficiency substantially, to 99% (CL 93–107%) for the present combined data for intervals of 1–2ms with the E-net, and with comparable figures of 98% (86–113) for the E-cloth. Third, while the increase of pulse interval above about 10ms causes reduced efficiency, the reduction is not great until intervals exceed about 70ms for E-nets; with the E-cloth the reduction in efficiency seems not quite so great and is not marked until >100ms. Fourth, while the video work with E-nets showed that fine netting appears to repel about 25% of the flies that would have collided with the grid, present results were surprising in showing that the fine netting seemed to attract roughly enough flies to compensate for this.

These points combine in various ways to suggest the appropriate electrocuting systems for various purposes. At one extreme is the sort of undemanding work involved in assessing the relative numbers of tsetse that alight on various visual baits [16,17,18]. For these one need not worry about the visual impact of the grid and its frame—the dominant visual effect is the bait itself. Relatively long pulse intervals of 15–30ms would ensure good catches while also reducing the frequency at which batteries need recharging. At the other extreme there are attempts to make the systems as perfect as possible. For example, with E-nets we now see that this involves wide trays, pulse intervals of 1–2ms, ultra-fine netting and the minimization of frame effects by positioning the frames far from the net, as in some of the present work, or perhaps using large nets ~1.5m wide [9] which ensure that on average the frames are far from most of the grid. If such large nets are used it would be safest to maintain the mJ/m^2^, perhaps by using a separate transformer to power different sections of the grid, as with the 1.5 x 3.3m E-nets used by [9], each half of which was separately powered. In attempting to increase the energy per pulse without committing to impracticable demands for total power, it would be sensible to reduce the pulse interval to 5ms since this seems unlikely to impact much on catches.

Between the above extremes is the range of devices that have been used for most of the studies with tsetse and other insects. While the efficiency and objectivity of these devices are certainly not as perfect as original considered, the devices have a track-record of proven usefulness in exposing the sort of important phenomena that cannot easily be studied by other methods. For this reason we repeat our previous recommendations [7] for the wider development and use of electrocuting devices against a greater variety of insects. In such matters it is necessary to recognise that present findings apply to robust and fast flying insects that are active in daylight, and may not apply in exactly the same way to those insects, such as mosquitoes, which are more delicate, slower flying and commonly active at night. For example, the sparking from grids is likely to be more visible to nocturnal insects. Presumably, this explains why field studies in Zimbabwe [7] and Kenya [19] found that reducing the spontaneous sparking at grids increased the catches of nocturnal mosquitoes, whereas such sparking seems to have no effect on tsetse catches [1]. For exploring the broader application of electrocuting devices, the present methods of catch comparisons, supplemented where possible by video studies, offer quick, simple and reliable aids to progress.

## Supporting Information

S1 Table Total catches of tsetse, biting and non-biting muscoids, and tabanids on the East and West sides of E-nets and E-cloth powered at pulse intervals of 1ms and 15ms, with an energy of 35mJ per pulse. (XLS)

S2 Table Catches of tsetse, biting and non-biting muscoids, and tabanids from E-nets and E-cloth powered by pulses at various test intervals, relative to catches at a standard interval of 15ms, using the one-site method. Various powers per pulse were used for the test and standard intervals. The data for tsetse are those used for Fig 4.
(XLS)

S1 Fig Circuitry for the latest type of transformer. The energy quoted refers to the energy per pulse discharged from the capacitors into the car coil.
(TIF)
