# Supplementary figures and images for: Efficacy of Electrocuting Devices to Catch Tsetse Flies (Glossinidae) and Other Diptera
Source: PLoS Negl Trop Dis. 2015 Oct 27;9(10):e0004169. doi: 10.1371/journal.pntd.0004169 (PMC4631496; doi:10.1371/journal.pntd.0004169)

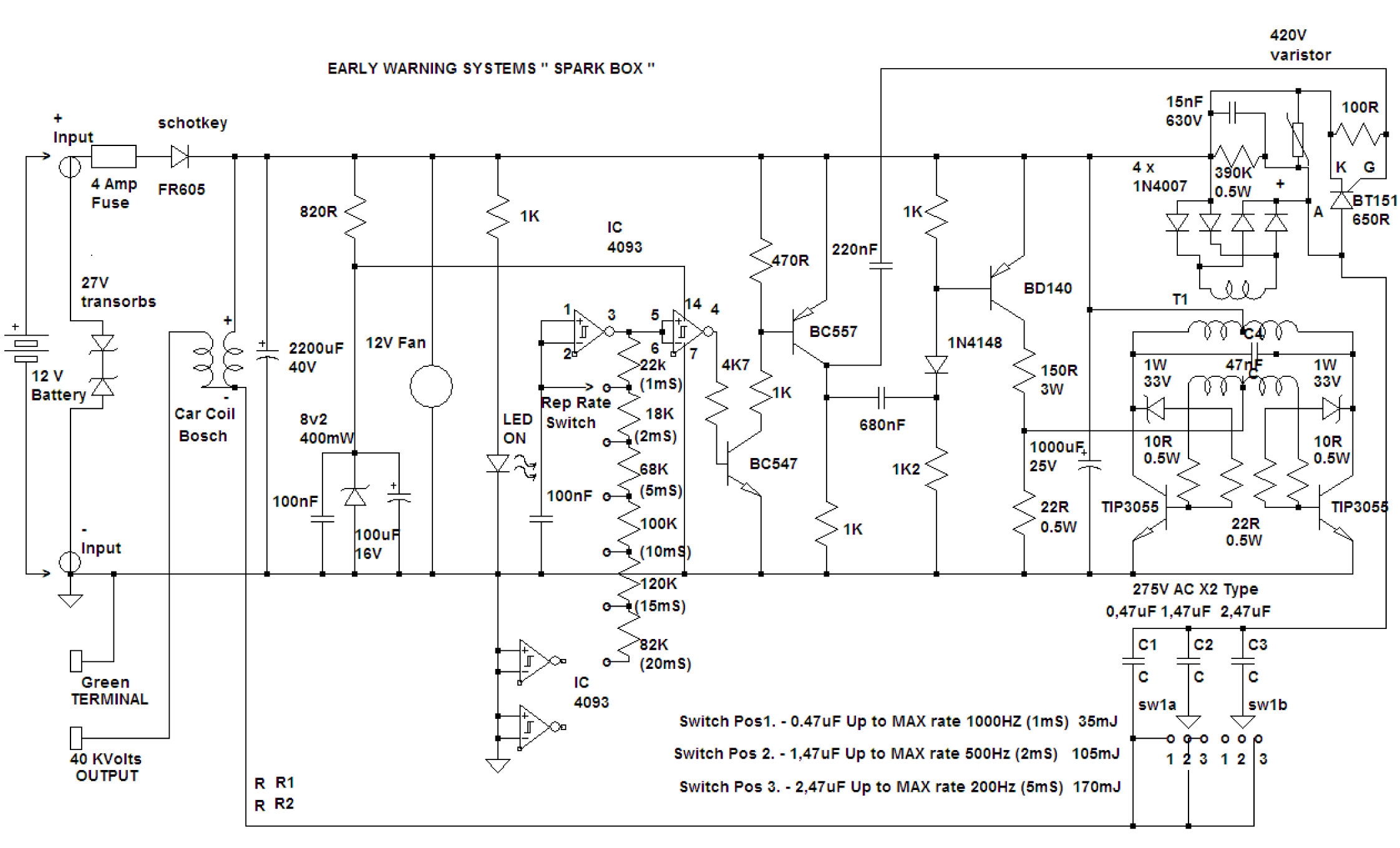

Supplement: S1 Fig — The energy quoted refers to the energy per pulse discharged from the capacitors into the car coil. (TIF) [file pntd.0004169.s003.tif]
